# Sustaining primary care teams in the midst of a pandemic

**DOI:** 10.1186/s13584-020-00434-w

**Published:** 2020-12-28

**Authors:** Erin E. Sullivan, Russell S. Phillips

**Affiliations:** 1 Department of Healthcare Administration, Sawyer Business School, Suffolk University, 73 Tremont Street, 5th floor, Boston, MA 02108 USA; 2 Center for Primary Care, Harvard Medical School, Harvard University, 635 Huntington Ave, Boston, MA 02115 USA; 3 Beth Israel Deaconess Medical Center, Beth Israel Lahey Health, Boston, MA USA

**Keywords:** Primary care, Teams, Interprofessional, Communication, Virtual work, COVID-19 pandemic

## Abstract

The challenges wrought by the COVID-19 pandemic on health systems have tested primary care clinicians, who are on the front lines of care delivery. To ensure the longevity of the primary care workforce, strong interprofessional teams are one important solution to alleviating burnout and increasing clinician and patient satisfaction, but the pandemic has demonstrated that the operating manual needs to be adapted for virtual work. Essential principles of primary care, including preventative care, communication and collaboration, and building strong relationships, can be applied to strengthen virtual primary care teams.

Despite differently organized health system structures, the United States and Israel share similar challenges when it comes to the primary care workforce. Both systems struggle to train and retain enough primary care clinicians, and predict that current shortages may grow within the next 10 years [1, 2]. These shortages are driven by a number of factors, including increasing demand due to aging populations; stagnant numbers of medical students selecting family medicine or other primary care-related specialties; and high rates of burnout leading to turnover or early retirement amongst practicing clinicians [2]. Over the past decade, interprofessional teams have become one important solution to primary care’s challenges, and practices have constructed teams with diverse members who can be responsive to their population [3]. Robust teams that include physicians, nurses, dieticians, pharmacists, and social workers strengthen and support primary care while alleviating burnout, increasing clinician satisfaction, and improving patient outcomes [4].

With the onset of the COVID-19 pandemic, however, stable, well-functioning primary care teams with reliable routines and processes were upended by new, virtual ways of working. Teams, used to huddling together in clinic, executing warm hand-offs to meet patient needs, and speaking in real-time, had to work virtually, often from home, with laptops, telehealth platforms, and their electronic medical record as their main patient care tools. Teams were further disrupted as members were reassigned, furloughed, took time off to care for family members, or left the workforce. The return of some in-person care during the continuation of the pandemic has meant that a small number of essential clinicians are in clinic- without their full team. Below, we share core team strategies that may help teams adapt to virtual work.

## Teams require preventive care for proper functioning

Team leaders need to monitor the team’s functioning, and individual levels of job satisfaction, on a regular basis to identify early symptoms or warning signs that can be addressed, and hopefully resolved, before they become major issues. For example, it can be hard to convey tone via email or chat, so messages that are intended to provide helpful feedback on patient care may be misinterpreted as being over-critical and unsupportive, leading to unintended job dissatisfaction. To avoid this, team members need to be queried on sources of dissatisfaction, and subject matter that is sensitive should be dealt with directly by spoken voice, using the telephone or another form of real-time interactive technology.

Developing and maintaining effective teams requires regular work on the mechanics of team functioning [5]. Teams should re-visit the team’s mission, identity, and purpose; redefine social norms; and reset roles and priorities. Investing the time in doing so will allow teams to consider what might no longer be working given current realities, and empower team members to suggest and implement improvements. A lack of clarity on mission, purpose, norms, and roles frequently results in unproductive teams with disconnected team members [6]. Teams should write down norms, and make a habit of reviewing or updating them at regular intervals to keep up with the changing environment (see Table 1).

**Table 1:** Team Norms for a Virtual Environment

Area of importance	In-person team	Virtual team
Meeting norms	Be present	Interruptions by children or pets are okay, please mute your mic
	No side conversations or comments	Use the chat box to engage during the meeting
Communication norms	Communicate in real-time as much as possible throughout the clinic day	When working remotely, please have designated team chat app open, and check at 90 min intervals
	Urgent tasks handed off immediately to the appropriate team member	Urgent tasks or needs should be communicated via phone or text to the appropriate team member; non-urgent tasks or needs can be sent via the inbox will be replied to within 24 h
Information sharing approach	Updates provided during daily team huddles and monthly all-clinic meetings	A designated “notetaker” will post important updates from huddles and all-clinic meetings in shared team collaboration space
	Agendas will be shared 24-h in advance of any meetings	Agendas will follow a structured template and be shared 48-h in advance with accompanying documentation
Collaboration tools	Team brainstorm and problem-solving sessions use the whiteboard in our team pod	Use virtual whiteboard for brainstorming and problem-solving in an asynchronous environment with a clear timeline for input
What else is important to the team?	Find joy in the work and each other	Take time to check-in with each other, ask how someone is doing and how you might be able to be supportive

## Effective communication and collaboration is a key principle for primary care teams

The inherent physical distance between co-workers in a virtual environment poses challenges with communication and collaboration. Proximity impacts what you know about your co-workers and disrupts the shared understanding that facilitates collaboration and coordination [7]. Geographic distance can make it difficult to coordinate between a team that used to sit in the same space, and work together on a daily basis. While technology can help teams communicate and collaborate in a virtual environment, too many virtual meetings or keeping up with asynchronous chat conversations can drive exhaustion, feelings of being overwhelmed, and disengagement [8].

Setting team norms specifically related to communication and collaboration is one way to ameliorate frequent, disjointed, and overwhelming modes of virtual communication. For example, re-visit your normal team meeting time and structure. Confirm if your weekly meeting time is still feasible for your team members given how individual schedules may have shifted to accommodate both personal and professional schedules. If the old time no longer works, find a new time that works for everyone. This signals that all team members are valued and that maintaining team connections is important. Further, adopting structured standing agendas for team meetings will often have the benefit of helping knowledge-sharing between team members [9].

## Virtual teams rely on strong relationships, just like primary care

Strong relationships between clinicians and patients is a hallmark of primary care, and patients often see the benefit in long-term, continuous relationships with a single clinician. Similarly, strengthening relationships amongst a virtual team will be beneficial to overall team outcomes. Clinicians have described the shift in camaraderie and increase in feelings of isolation at work due to COVID; one primary care physician explained: “Before the pandemic, we had a central team room where other providers would hang out and write notes. Then you’d go see patients in person, you’d come back, and we would talk to each other. We would advise each other, and it was a really fun collegial experience. Now when we are in clinic, only a small number of us are there in person; we can’t sit in our team room; and we don’t talk to each other or see each other. I think that loses a lot. We could pick up the phone and call one another, but that just seems to reinforce the personal disconnection we are all feeling.”

Taking the time to continue to build relationships amongst team members strengthens trust, psychological safety, and connection, all which drive collaboration. One simple way of doing this is to start meetings with personal/professional check-ins from every team member. This helps combat feelings of isolation that come with virtual work. Remote celebrations of team accomplishments- of “wins” both big and small- boost feelings of collective efficacy, or “this team can succeed.” These feelings can help to improve connection and collaboration [10]. Teams that socialize remotely benefit from building rapport and creating empathy amongst team members. Finally, leaders should take opportunities during 1:1 conversations to check-in on an individual’s well-being and if there is anything the leader can do to better support individuals during challenging times.

Now is a time to work on strengthening team function, whether the team is operating in the same physical space, or virtually. Brief surveys to measure team function can be used as a “pulse-check” by leaders to gather more information on how things are going and to drive discussions about opportunities for innovation and improvement. Just as practices rapidly adopted telemedicine to connect with patients who are unable to meet in person, we should also adopt new approaches that will enhance patient engagement and satisfaction. Having high functioning, collaborative teams works best for patients and also for team members.

## Data Availability

Not applicable.
